# Elucidating the preventive and therapeutic effects of cardiac telocytes paracrine microRNAs on ischemic heart disease

**DOI:** 10.3389/fcvm.2025.1540051

**Published:** 2025-04-01

**Authors:** Hugang Jiang, Yan Tang, Ai Liu, Chunzhen Ren, Wenyan Lin, Kai Liu, Xinke Zhao, Yingdong Li

**Affiliations:** ^1^ Department of Traditional Chinese and Western Medicine, Gansu University of Chinese Medicine, Lanzhou, Gansu, China; ^2^ Daytime Diagnosis and Treatment Center, Gansu Provincial People’s Hospital, Lanzhou, Gansu, China; ^3^ Cardiovascular Clinical Medical Center, Affiliated Hospital of Gansu University of Chinese Medicine, Lanzhou, Gansu, China

**Keywords:** cardiac telocytes, paracrine secretions, endothelial cells, crosstalk mechanism, IHD

## Abstract

Telocytes (TCs), a newly identified type of mesenchymal cell since 2010, possess substantial potential in maintaining tissue homeostasis, orchestrating organ development, and facilitating tissue regeneration. Their distribution in blood, the adventitia of blood vessels, and the intima implies a close association with vascular function. Ischemic heart disease (IHD), a significant challenge in cardiovascular disease, is characterized by the occlusion of major vessels, obstruction of collateral circulation, and disruption of the capillary network—pathological features closely linked to endothelial cell damage. Myocardial tissue is rich in cardiac telocytes (cTCs), which, following myocardial injury, can secrete numerous miRNAs that promote angiogenesis, including miR-let-7e, miR-10a, and miR-126-3p. This indicates that cTCs may have therapeutic potential for IHD. The primary mechanism by which cTCs-derived exosomes exert paracrine effects is through reducing endothelial cell injury, suggesting that enhancing the production of cTCs could offer a novel therapeutic approach for treating IHD.

## 1 Introduction

In recent years, with the rapid development of evidence-based medicine and interventional cardiology, scholars have been increasingly recognizing the previously unappreciated significance of ischemic heart disease (IHD) (1–3). IHD is damage to the heart muscle due to an imbalance between coronary blood flow and the needs of the heart caused by changes in coronary circulation. Both coronary arteries and coronary microvessels have the potential to significantly influence the perfusion of myocardial tissue. Furthermore, coronary microvascular injury is a significant contributor to IHD due to their crucial role in the exchange of metabolic substances. According to the study, in individuals with stable angina pectoris, the injury rate of coronary microvessels without obstructive coronary artery disease was 67%, and women had a significantly higher frequency than men (4). This may be attributed to several factors. Firstly, the diameter of coronary microvessels in women is relatively smaller than that in men, rendering them more vulnerable to stenosis or obstruction. Secondly, the decline in estrogen levels following menopause elevates the risk of coronary microcirculation disorders. Additionally, women are more susceptible to psychological disorders such as anxiety and depression (5).

The fundamental pathological changes associated with IHD include myocardial tissue hypoperfusion caused by coronary artery stenosis, collateral circulation disturbance following coronary artery occlusion, and coronary microvascular dysfunction (6). Myocardial tissue hypoperfusion-induced vasoactive factors can lead to coronary spasm, facilitate endothelium-dependent intra-coronary thrombosis, reduce coronary artery barrier function, and aggravate the inflammatory response and myocardial fibrosis following myocardial ischemia (7). Furthermore, endothelial cell injury can further exacerbate coronary arteriosclerosis, which is the deterioration of myocardial perfusion and the cause of IHD (8). Endothelial cells play a pivotal role in the pathogenesis of IHD, being the most significant cell component of the vascular intima (9). Hence, endothelial cell injury is a significant cause of IHD, and preserving the normal structure and function of endothelial cells is crucial for preventing and treating IHD, particularly IHD resulting from coronary microvascular dysfunction.

cTCs are a novel type of interstitial cell with unique morphology and important physiological functions. cTCs are widely distributed in various layers of the heart, including the epicardium, myocardium, and endocardium. Particularly in the subendocardial layer, cTCs exhibit a distribution characteristic with a relatively large number and complex morphology, and form an extensive cell interaction network among target cells (10).

cTCs provide mechanical support for the cardiac tissue through their complex protrusion network, helping to maintain the structural integrity of the heart. cTCs contact other cells (such as cardiomyocytes and endothelial cells) through telopodes (Tps), regulate the activity of cell membrane Ca^2+^ channels, and participate in the electrophysiological activities and functional regulation of the heart (11). Tps are the most important structural markers of cTCs and also the structural basis for cTCs to exert paracrine functions. They are bead-like pseudopodia extending from the body of cTCs, traversing the intercellular space. This structural formation serves as a vital basis for cTCs to modulate the functions of neighboring cells. cTCs establish direct or indirect connections with stem cells in the cardiac stem cell niche. They may participate in the regulation of stem cell proliferation and differentiation by releasing small vesicles secreted by cells (containing various proteins, RNA, and lipids) and multiple cytokines, such as vascular endothelial growth factor (VEGF), fibroblast growth factor (FGF). They are promoting the repair and regeneration of cardiac tissue (12). cTCs may even be involved in the cardiac immune response, modulate inflammatory reactions, and uphold the homeostasis of cardiac tissue (13–17).

Studies have demonstrated that during the angiogenesis phase subsequent to an acute myocardial infarction, the number of cTCs significantly increases in the border zone of the infarcted area. The method of injecting cTCs (a quantity of 10⁶) into the infarct border zone after myocardial infarction can observe the reduction of myocardial infarction area, increased angiogenesis, decreased myocardial fibrosis, and improved cardiac function after 14 weeks (18–21). cTCs-secreted miRNAs, such as miR-let-7e, miR-10a, and miR-126-3p, were found to significantly promote endothelial cell proliferation, migration, and tubulation in a recent study (22). cTCs play a beneficial role in cardiac angiogenesis and endothelial cell repair and may be a key target for preventing and treating IHD. Excitingly, a study directly confirms that cTCs can inhibit the apoptosis of cardiac endothelial cells by silencing cdip1 through targeted exosomal miRNA-21-5p, thereby enhancing angiogenesis following myocardial infarction. This provides a solid basis for this paper (23). Furthermore, six studies have confirmed that TCs possess the capability to effectively improve circulation disorders (24–29). Though these studies weren't done directly on the heart, they were separately carried out on human lung tissue, skin wound healing models, trigeminal ganglia perivasculature, pig dermal microvascular units, skeletal muscle interstitium, and camel epididymis. Their results strongly back the fundamental viewpoint of this paper.

## 2 cTCs-a unique type of myocardial interstitial cell

### 2.1 Morphological differences between cTCs and TCs in other tissues

Compared with TCs in the intestine and skin, cTCs, due to being in the heart tissue that is under continuous mechanical stress and needing to withstand greater tension and provide effective mechanical support during myocardial contraction and relaxation, usually have relatively thicker Tps that are relatively shorter in length. Moreover, to ensure the efficient transmission of mechanical signals and the maintenance of cellular stability in the complex mechanical environment of the heart, the beaded structure of their Tps may be more compact. The cell bodies of cTCs are relatively smaller and more regular, usually oval or spindle-shaped. This shape helps the cells to be closely arranged between the myocardial fibers, so as to better perform their functions of mechanical support and signal transduction (30–32).

However, in intestinal tissue, the cell bodies of TCs tend to be relatively elongated and exhibit a slight spindle shape, which is an adaptive feature to accommodate the intestinal peristalsis and the complex tissue architecture (33, 34). In lung tissue, TCs often display a more flattened morphology, enabling them to attach more effectively to the alveolar surface or establish tight junctions with adjacent cells (35). In neural tissue, the Tps of TCs are typically shorter and sparsely distributed, primarily forming local networks around the neuronal cell bodies or axons (36). Within the liver, the protrusions of TCs are usually longer and more extensively distributed, traversing the hepatic lobules and playing a significant role in the metabolic and immune regulatory functions of the liver (37, 38). In skin tissue, the branches of TCs protrusions are relatively fewer in number and more regular in pattern, presenting an orderly arrangement that helps maintain the skin's elasticity and barrier function (39). In pancreatic tissue, the branches of TCs protrusions are relatively complex and may engage in close interactions with islet cells and ductal cells, thereby participating in the regulation of the pancreas's endocrine and exocrine functions (40). In immune tissue, TCs can interact with immune cells via immunological synapses, actively participating in the transmission of immune signals and the regulation of immune responses (41). Meanwhile, in reproductive tissue, TCs may form specific connections with germ cells, providing essential support and protection for the development and maturation of these cells (42).

### 2.2 Differences between cTCs and other cardiac interstitial cells

The myocardium is composed of a diverse assortment of interstitial cells, each of which plays a crucial role in both the physiological and pathological facets of cardiac function (43). Take fibroblasts, they provide vital mechanical support and contribute to the structural organization of cardiomyocytes, thus maintaining the heart's normal morphology and functionality (44). cTCs, distinguished by their elaborate network of protrusions, play a significant role in facilitating intercellular signaling, thereby helping to maintain cardiac electrical stability (14). Mesenchymal stem cells, with their remarkable plasticity, can differentiate into cardiomyocytes and endothelial cells following myocardial injury, thereby facilitating the repair and regeneration processes (45). Lymphocytes are essential for modulating the cardiac immune response, as they help to suppress excessive inflammation and protect against further tissue damage (46). Endothelial cells, through the secretion of growth factors, promote angiogenesis, increasing myocardial blood flow and facilitating functional recovery (47). Nevertheless, these interstitial cells display considerable heterogeneity in terms of their abundance, spatial distribution, immune profiles, and primary functions, as elaborated in Table 1.

**Table 1:** The differences between cTCs and other cardiac interstitial cells.

Number	Aberration	Designation	Cell proportion	Cell location	Cell surface marker	Cell intracellular marker	Cell secretory marker	Primary biological function
1	cTCs	Cardiac telocytes	0.5%–1%	Widely distributed in the myocardial interstitium	c-Kit, CD34, PDGFR-α/β	α-Actin, Cx43	VEGF, HGF	1. Maintain the structural integrity 2. Regulate the electrophysiological activities and functional 3. Promote the repair and regeneration of cardiac tissue 4. Participate in the immune aninflammatory response
2	cFs	Cardiac fibroblasts	70%	Mainly located between cardiomyocytes	vimentin, fibronectin	Prolyl hydroxylase, α-SMA	MMPs, TIMPs, CollagenI, CollagenIII	1. Synthesize and secrete extracellular matrix 2. Participate in the repair process of cardiac injury 3. Regulate the growth and differentiation of cardiomyocytes
3	cMSCs	Cardiac mesenchymal stem cells	In small amounts	1. Present around the small blood vessels in the heart 2. Near the damaged myocardial area	CD73, CD90, CD105	Oct4, Nanog	HGF, Chemokines	1. Undertake directional differentiation 2. Regulate the local microenvironment 3. Inhibit inflammatory responses 4. Promote angiogenesis 5. Participate in the repair and regeneration of cardiac tissue
4	cSMCs	Cardiac smooth muscle cells	17%	In the walls of cardiac blood vessels and valve tissue	α-SMA	Calmodulin, Actin, Myosin	Collagen, Elastin	Be involved in the composition of blood vessel walls and vascular remodeling
5	cECs	Cardiac endothelial cells	30%	The inner surface of the cardiac blood vessels and microvascular network	CD34, eNOS, VE-cadherin	Connexin, Receptor tyrosine kinase	NO, PGI₂, ET-1	1. Constitutes the endothelial barrier of blood vessels 2. Participates in the regulation of vasodilation and vasoconstriction 3. Promotes the balance between blood coagulation and fibrinolysis 4. Participates in the process of angiogenesis
6	cNCs	Cardiac neuron cells	In small amounts	In the plexus of the heart	NSE, Syn	NeuroD	Acetylcholine, Norepinephrine	1. Participate in the electrophysiological activities of the heart 2. Regulate the rhythmic activities of the heart
7	cMs	Cardiac macrophage cells	In small amounts	The interstitium of the heart, around blood vessels, at inflammatory sites, and in areas of injury	CD68	Lysosome - associated membrane protein	TNF-α, IL-1β, IL-6	Participate in the immune defense of the heart, tissue repair and the formation of myocardial fibrosis
8	cLs	Cardiac lymphocyte cells	In small amounts	Mainly present around the blood vessels, lymphatic vessels in the heart and at inflammatory sites	CD3, CD19		IFN-*γ*, IL-2	Participate in the immune response of the heart
9	cMCs	Cardiac mast cells	In small amounts	Mainly distributed around the cardiac vessels, in the myocardial interstitium, on the cardiac valves, and in areas of allergic reactions	PGD₂, Fc*ε*RI		Histamine, Leukotriene, PGD₂	Participate in the allergic reaction and inflammatory response of the heart

This table provides a summary of the various types of interstitial cells in the heart, including their primary distribution locations, surface markers, intracellular markers, secretory markers, and main biological functions. (c-Kit, proto-oncogene protein c-kit; CD34,cluster of differentiation 34; PDGFR-α/β, platelet-derived growth factor receptor alpha/beta; Cx43, connexin 43; VEGF, vascular endothelial growth factor; HGF, hepatocyte growth factor; α-SMA, alpha-smooth muscle actin; MMPs, matrix metalloproteinases; TIMPs, tissue inhibitors of metalloproteinases; CD73, cluster of differentiation 73; CD90, cluster of differentiation 90; CD105, cluster of differentiation 105; Oct4, octamer-binding transcription factor 4; Nanog, nanog homeobox transcription factor; Calmodulin, calcium-modulated protein; eNOS, endothelial nitric oxide synthase; NO, nitric oxide; PGI₂, prostaglandin I2; ET-1, endothelin-1; NSE, neuron-specific enolase; CD68, cluster of differentiation 68; TNF-α, tumor necrosis factor-alpha; IL-1β, interleukin-1beta; IL-6, interleukin-6; CD3, cluster of differentiation 3; CD19, cluster of differentiation 19; IFN-γ, interferon-gamma; IL-2, interleukin-2; PGD₂, prostaglandin D2; Fc*ε*RI, high-affinity IgE receptor).

### 2.3 The typical structural characteristics of cTCs

**Figure 1:**
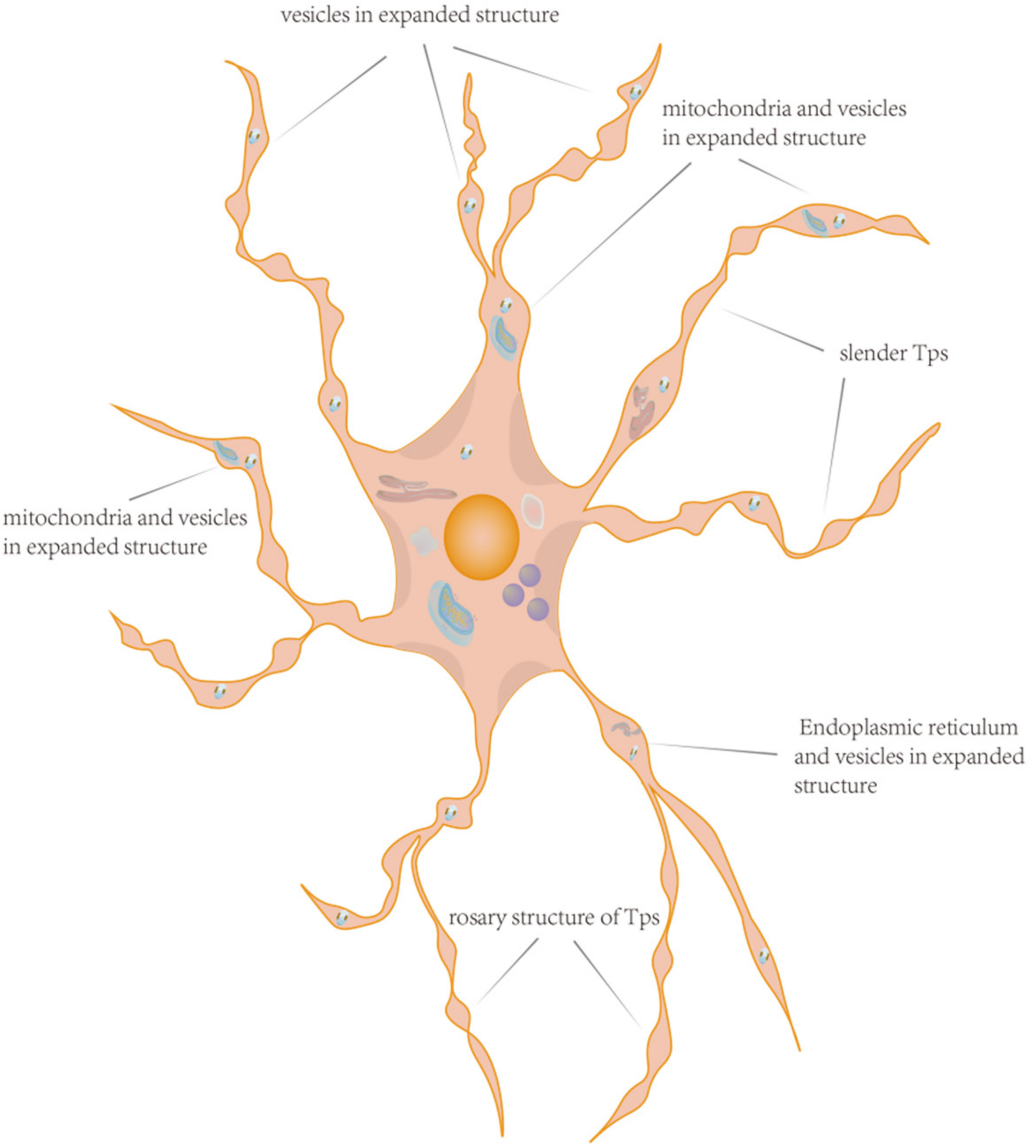
The pattern diagram of cTCs. This schematic illustration of a cTCs vividly displays its morphological features, featuring an irregular cell body with Tps extending outward. Within the cell body reside typical organelles such as the nucleus, mitochondria, and endoplasmic reticulum. The Tps exhibit a distinctive pattern of bead-like expansions interspersed with narrow segments. Notably, the expanded portions are rich in organelles, and the Tps also display branching characteristics. The distinctive structure of cTCs underpins their ability to perform complex biological functions.

The distinctive physical and structural characteristics of cTCs, in contrast to other cells, are their relatively small cell bodies (ranging from 9 to 15 μm) and the multiple slender Tps that extend from the cell body, with lengths reaching up to 100 μm. Among these features, the most significant structural attribute that sets cTCs apart from other cell types is the presence of these unique Tps. Depending on the number of Tps, the cell body of cTCs can exhibit different shapes, such as pear-shaped, fusiform-shaped, triangle-shaped, star-shaped (48). The nucleus makes up approximately 25% of the cell volume and contains clusters of heterochromatin attached to the nuclear membrane (16, 49). The cytoplasm contains many subcellular structures including mitochondria, golgi apparatus, endoplasmic reticulum, and cytoskeleton (50–52). Each Tps has a length of approximately tens to hundreds of micrometers and a width of around 0.10 ± 0.05 micrometers. Due to the extremely narrow width of Tps, it is challenging to observe under ordinary optical microscopes their extension into the surrounding matrix and the formation of connections with other cells. When Tps are observed under an electron microscope, their rosary-like structure becomes apparent. This structure is characterized by alternating regions of expansion and constriction. Additionally, Ca^2+^-release units andorganelles such as endoplasmic reticulum, mitochondria, and vesicles were observed at the level of the dilations (53), that were beneficial for the function of mitochondria and endoplasmic reticulum, and also plays a positive role in the transport of exosomes and vesicles (10), as shown in Figure 1.

### 2.4 The special molecular markers of cTCs

cTCs exhibit certain differences in molecular markers compared with TCs in other tissues. These differences reflect their specific functions and adaptabilities in different tissue microenvironments. Cluster of differentiation 34(CD34), c-Kit, and platelet-derived growth factor receptor α/β (PDGFR-α/β) are common surface markers of cTCs (54, 55). In the heart, the expression of CD34 helps cTCs interact with other cells (such as cardiomyocytes and vascular endothelial cells), participating in the repair and regeneration of cardiac tissue. c-Kit may be involved in regulating the growth and differentiation of cardiomyocytes, as well as the regeneration and repair processes of cardiac tissue. The PDGFR-α/β signaling pathway plays an important role in cardiac development, myocardial hypertrophy, and the occurrence and development of cardiac diseases. α-Actin and connexin 43 (Cx43) are markers within the cytoplasm of cTCs. α-Actin may be involved in regulating the mechanical contraction function of cells, while Cx43 is involved in intercellular electrical signal transduction, which is crucial for maintaining normal heart rhythm and electrophysiological activities in the heart (56). VEGF and HGF are important secretory molecular markers of cTCs (22). VEGF can promote angiogenesis and increase vascular permeability, which is of great significance for maintaining the blood supply of the myocardium and the normal function of the heart. HGF has various biological functions, such as promoting cell proliferation, migration, and differentiation, and may be involved in regulating the growth and repair of cardiomyocytes, as well as the reconstruction of the myocardial interstitium. In intestinal TCs, the expression of vimentin is relatively significant. Integrin β4 is an important surface marker of skin TCs (57). Neurofilaments are one of the important intracellular markers of TCs in nerve tissue (58). Cytokeratins are the main structural proteins of hepatic TCs. In pulmonary TCs, more surfactant-related proteins may be secreted, while renal TCs may secrete molecules related to renal physiological functions, such as renin (59).

### 2.5 Isolation and purification of cTCs

Enzymatic digestion stands as the predominant technique employed for isolating cTCs (60). Initially, an appropriate sample of heart tissue is procured and subsequently minced into smaller portions. These tissue pieces are then transferred into a digestion medium comprising 1–3 mg/ml of type I collagenase, where the digestion process unfolds at 37℃ over a duration of 30–60 min. Following this, the digested tissue suspension is filtered using a mesh (commonly ranging between 100–200 μm) to yield a single-cell suspension enriched with heart cells. Various methodologies are employed for the purification of cTCs, notable among which are density gradient centrifugation, immunomagnetic bead separation, and flow cytometric sorting. Immunological markers such as c-Kit, CD34^+^, and PDGFR-α are frequently utilized in identifying cTCs. Leveraging these specific immune markers, the purification process culminates in the acquisition of cTCs with a high degree of purity.

## 3 The forms of information transmission between cTCs and other cells

The cardiac cTCs establish extensive connections with various types of cells, including other cTCs, endothelial cells, cardiac myocytes, cardiac fibroblasts, and cardiac stem cells, via their cell bodies and thin Tps (61, 62). cTCs can form a three-dimensional network of interstitial tissue by interacting with themselves and other cells. This enables them to directly or indirectly transmit information between cells, which is crucial in facilitating signal transmission.

### 3.1 The direct contact form between cTCs and other cells

Electronic tomography technology reveals three types of direct contact: point contact, electron-density nanostructure, and planar contact (11). In the human myocardium, cTCs engage in direct interactions with a diverse array of cell types. These comprise cardiomyocytes, which are responsible for myocardial contractility and relaxation. Fibroblasts, pivotal in maintaining cardiac extracellular matrix homeostasis and structural integrity. Endothelial cells, which line the vascular lumen and regulate vasomotor and permeability functions. Cardiac stem cells, endowed with self-renewal and multilineage differentiation potential. Mast cells, which play a significant role in cardiac immune surveillance and inflammatory responses (63, 64). Studies have confirmed frequent and close connections between vascular endothelial cells and cTCs through three different mechanisms (12, 65). Jeremiah Bernier Latmani et al. discovered that cTCs were extensively integrated into the subepithelial zone. Quantification of their cellular interactome indicated that the majority (around 70%) of direct cell contacts were either with other cells at the tip of the small intestinal villus. These findings position cTCs as perivascular cells uniquely situated at the villus tip, playing a crucial role in maintaining a polarized VEGFA signaling domain and promoting fenestrations within the nutrient-absorbing intestinal blood vessels (66).

### 3.2 The indirect contact form between cTCs and other cells

cTCs act as intermediaries to enable communication between cells by facilitating exo- and endocytosis. During exocytosis, vesicles containing complex bioactive components act as important carriers for signal transmission between cTCs and other cells (67, 68). The vesicles secreted by cTCs can be categorized into three types according to their diameters: exosomes (45 ± 8 nm), ectosomes (128 ± 28 nm), and multivesicular cargo (1 ± 0.4 µm) (12, 69). Despite structural differences, these vesicles share similar biological functions. When the vesicle membrane degrades within the extracellular matrix, a diverse range of biological information is released and diffuses into the heart tissue, thereby affecting its function (70–72). cTCs also have endocytosis function, allowing them to uptake vesicles secreted by cardiac stem cells, cardiac myocytes, and endothelial cells and facilitate the retrogressive information transmission (12, 72). This phenomenon of information feedback has been experimentally verified using ink.

## 4 Connection between TCs and blood vessels

Studies have demonstrated an intimate relationship between TCs and blood vessels. TCs in circulating blood can adhere to endothelial cells in arteries (73). Concerning the origin of TCs in the blood, the studies confirmed that they are derived from the bone marrow (53, 74, 75). Three distinct characteristics were observed in bone marrow telocytes: relatively small cell bodies, the presence of 1–3 Tps, and extensive connections with homologous cells (76–78). The presence of TCs in the bone marrow is in harmony with a significant number of bone marrow stem cells. Furthermore, cardiac stem cells are located in the epicardial subcardium, the same region where cTCs reside. This suggests that cTCs may have the potential to function as cardiac stem cells or play an important role in regulating the function of cardiac stem cells (79, 80). Recent studies have suggested that the tunica externa's cTCs exhibit certain markers that are characteristic of mesenchymal stem cells. Furthermore, these cTCs have been found to facilitate the exchange of information between stem cells of various tissues such as cardiac progenitor cells, sub-epithelial lung stem cells, skeletal muscle stem cells, and skin stem cell clusters, among others, through the release of shedding vesicles (81, 82). Cantarero et al. discovered that TCs are present around the connective tissue of arteries, veins, and capillaries. The distribution of TCs and their Tps is parallel to that of vascular smooth muscle cells (83). At the end of Tps, there is a swollen structure containing rough endoplasmic reticulum, mitochondria, and ribosomes. This structure may facilitate improved better signal transmission within the “Tps-Tps” complex (84, 85). The tunica externa of blood vessels contain TCs which do not make direct contact with smooth muscle cells or elastic fiber membranes. However, the desmosome structure of cells is widely present between “Tps-Tps” structures which helps TCs to form a three-dimensional network structure in the tunica externa (11, 86). There are TCs present in the sub-endothelial layer of the intima of the inferior vena cava. A significant amount of fibrous tissue accumulates around the TCs in the intima, which distinguishes them from those found in the artery (53).

cTCs, endothelial progenitor cells, and cardiac stem cells all play crucial roles in maintaining vascular function and promoting angiogenesis. Moreover, these three cell types share certain similarities while also exhibiting distinct differences. The cell bodies of all three are relatively small, facilitating their flexible movement within the interstitial tissue and enabling them to interact with other cells. Additionally, all three possess protrusion structures that allow them to exchange information with surrounding cells and the environment, thus playing a vital role in tissue repair and regeneration (87). However, there are notable differences among them. cTCs not only participate in regulating vascular permeability and promoting angiogenesis but also play a significant role in maintaining the electrophysiological function of the heart. Endothelial progenitor cells primarily focus on peripheral angiogenesis and the restoration of vascular function by differentiating into endothelial cells (88). Mesenchymal stem cells, on the other hand, regulate the microenvironment of the entire tissue by influencing the proliferation, differentiation, and metabolism of surrounding cells (78). In summary, TCs population acts as helper cells for stem cells and capillaries, they play a crucial role in maintaining homeostasis after vascular injury by regulating angiogenesis, regeneration, and repair processes.

## 5 Potential mechanisms and targets of cTCs for IHD

**Figure 2:**
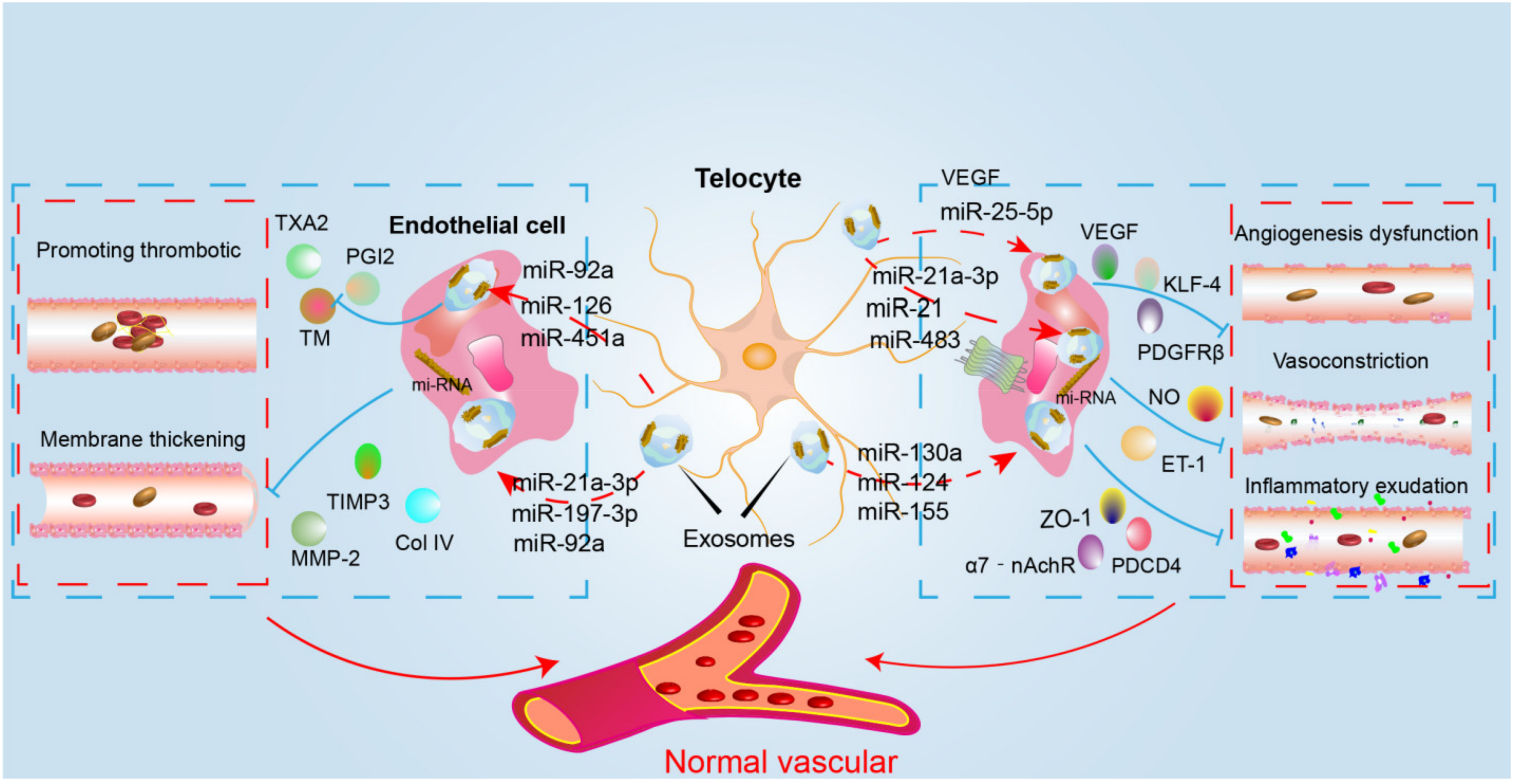
The mechanism diagram of cTCs intervention in CMVD. The figure depicts the mechanism through which cTCs enhance vascular function via paracrine secretion of diverse miRNAs. Specifically, they secrete miR-92a, miR-126, miR-451a, and others to regulate factors such as TXA2 and PGI2, thereby inhibiting thrombosis. Additionally, the secretion of miR-92a, miR-197-3p, miR-21a, and related miRNAs modulates TIMP3, MMP2, ColIV, and other molecules to suppress vascular remodeling. cTCs also secrete miR-25-5p to regulate VEGF and KLF-4, promoting angiogenesis. Furthermore, the secretion of miR-21a, miR-483, and other miRNAs adjusts NO and ET-1 levels, modulating vasodilation and vasoconstriction. Lastly, the secretion of miR-124, miR-155, miR-130a, and other miRNAs regulates ZO-1, PDCD4, and other factors, thereby influencing vascular permeability.(VEGF, vascular endothelial growth factor; PDGFR-β, platelet-derived growth facter receptor; KLF-4, Krüppel-like factor4; TM, thrombomodulin; TXA2, thromboxaneA2; PGI2, prostaglandin-I-2; ET-1, endothelin-1; ZO-1, zonula occludens protein 1; NO, nitric oxide;PDCD4, programmed cell death 4; 7-nAchR, α7 nicotinic acetylcholine receptor; TIMP3, tissue inhibitors of metalloproteinase 3; MMP-2, matrix metalloproteinase 2).

Several miRNAs, lncRNAs, mtDNA, and proteins—including VEGF, nitric oxide synthase 2 (NOS2), collagen type III alpha 1 (COL3A1), slit homolog 3 (SLIT3), follistatin (FST), neuronatin (NNAT), and protocadherin 17 (PCDH17)—serve as key active substances in the paracrine signaling of circulating tumor cells (cTCs), as shown in Figure 2 and Table 2. Although Dongli Song etal. found that TC17000728.hg.1, TC06001978.hg.1, TC08000302.hg.1, TC07001784.hg.1 and TC03003114.hg.1 were the most five expressed lncRNAs in cTCs (89), their biological functions have not been reported, so they will not be further discussed in this article. It is noteworthy that numerous studies have confirmed that mitochondrial DNA (mtDNA) plays a pivotal role in the communication between cTCs and endothelial cell (90). In future research, targeting the mitochondria, mtDNA and lncRNA may provide a new prognostic biomarker for diseases.

**Table 2:** The mechanisms and targets of improving IHD by cTCs.

Biological effection	Type of miRNA	Potential target	Related signal pathway	Pathological characterization	Bibliographic references
Promoting angiogenesis	miR-25-5p	Cdip1	–	Inhibit apoptosis	(52)
	–	NOS2	–	Maintaining cardiac angiogenesis	(18)
	–	VEGF	–	Maintaining cardiac angiogenesis	(18)
	–	PDGFR-β	–	Maintaining cardiac angiogenesis	(51, 53, 54)
	–	KLF-4	–	Cells proliferation and differentiation	(51, 53, 54)
Anti-thrombotic	miR-92a	KLF2, TM	–	Anticoagulant	(55, 56)
	miR-21, miR-409-3p, miR-432 and miR-150	–	–	Anticoagulant	(57–59)
	miR-126	–	–	Thrombosis	(60, 61)
	miR-451a	TF, Fxa, vWF, ATIII, TFPI, t-PA	IL6R/STAT/TF	Anticoagulant	(62)
	miR-26a-5p	TXA2, PGI2, ET-1, Ang II	PI3K/AKT	Inhibit apoptosis	(63)
	miR-301a/miR-454	HIF-1α, PPAR-α, ET-1, PAI-1	–	Anticoagulant	(64)
Inhibition of inflammatory exudation	miR-130a	HOXA5	–	Enhanced the permeability	(65, 66)
	miR-124	IL-4, IL-13	IL-6/TNF-α	Anti-inflammatory	(67)
		ROS	PI3K/AKT	Inducing apoptosis, Enhanced the permeability	(68)
	miR-155	ZO-1, eNOS	–	Enhanced the permeability, vasodilation	(69, 70)
		–	JAK/STAT	Anti-inflammatory	(71)
		C/EBP β	–	Enhance the inflammatory response	(72)
	MiR-499	PDCD4	NF-kB/TNF-α	Enhance the inflammatory response	(73)
		α7-nAchR	–	Enhance the inflammatory response	(74–76)
Regulatio of microvascular diastolic and systolic function	miR-21a-3p	eNOS	–	Vasodilation	(80)
	miR-21	ET-1, eNOS	MAPK/ET-1	Contraction function	(81)
			PTEN/AKT/eNOS	Vasodilation	(81)
	miR-483	ET-1	–	Vasodilation	(82)
	miR-92a/miRNA-1	ET-1	–	Contraction/Vasodilation	(83)
	miR-124, miR-34c	PGI2	JAK2/STAT3	Vasodilation	(84)
Inhibition of vascular remodeling	miR-197-3p	TIMP3, vWF	–	Collagen deposition, Thrombosis	(88, 89)
	miR-21a-3p	MMP-2	PI3K/AKT/mTOR	Matrix degradation	(80)
	miR-92a	α-SMA, SMTN, CALP, FN, OPN, TSP	–	Phenotypic transformation,Arteriosclerosis	(90)
	miR-155-5p	VEGFa, FGF2, MMP9, SOCS1	SOCS1	Vascular remodeling	(91)

This table presents a summary of the key miRNAs involved in promoting angiogenesis, inhibiting thrombosis, suppressing inflammatory exudation, regulating vasodilation and vasoconstriction, and inhibiting vascular remodeling. Additionally, it offers an overview of their downstream targets, corresponding signaling pathways, and principal biological functions.(Cdip1, Cdip1 gene; NOS2, nitric oxide synthase 2; VEGF, vascular endothelial growth factor; PDGFR-β, platelet-derived growth facter receptor; KLF-4, Krüppel-like factor4; KLF2, Krüppel-like factor 2; TM, thrombomodulin; TF, tissue factor; Fxa, actived coagulation factor X; vWF, von Willebrand factor; ATIII, antithrombin III; TFPI, tissue factor pathway inhibitor; t-PA, tissue plasminogen activator; IL-4, interleukin-4; IL-6, interleukin-6; IL-1, interleukin-13; IL6R, interleukin 6 receptor; JAK/STAT, Janus kinase/signal transducers and activators of transcription; JAK2/STAT3, Janus kinase 2/signal transducers and activators of transcription 3; TXA2, thromboxaneA2; PGI2, prostaglandin-I-2; ET-1, endothelin-1; Ang II, angiotesin- II; PI3K/AKT, phosphatidylinositol 3 kinase protein kinase B; HIF-1α, hypoxia inducible factor-1α; PPAR-α, peroxisome proliferators-activated receptor alpha; PAI-1, 1-type plasminogen Inhibitor; HOXA5, Homeobox A5; TNF-α, tumor necrosis factor-α; ROS, reactive oxygen species; ZO-1, zonula occludens protein 1; eNOS, endothelial nitric oxide synthase; C/EBP β, enhancer-binding proteins β; PDCD4, programmed cell death 4; NF-kB, nuclear factor-k-gene binding; 7-nAchR, α7 nicotinic acetylcholine receptor; MAPK, mitogen-activated protein kinase; PTEN, gene of phosphate and tension homology deleted on chromsome ten; TIMP3, Tissue Inhibitors Of Metalloproteinase 3; MMP-2, matrix metalloproteinase 2; MMP9, matrix metalloproteinase 9; mTOR, mammalian target of rapamycin; α-SMA, α-smooth muscle actin; SMTN, smoothelin; CALP, calpactin; FN, fibronectin; OPN, osteopontin; TSP, total serum protein; VEGFa, vascular endothelial growth factor a; FGF2, fibroblast growth factor 2; SOCS1, suppressors of cytokine signaling 1).

### 5.1 The mechanism of cTCs promoting angiogenesis

**Figure 3:**
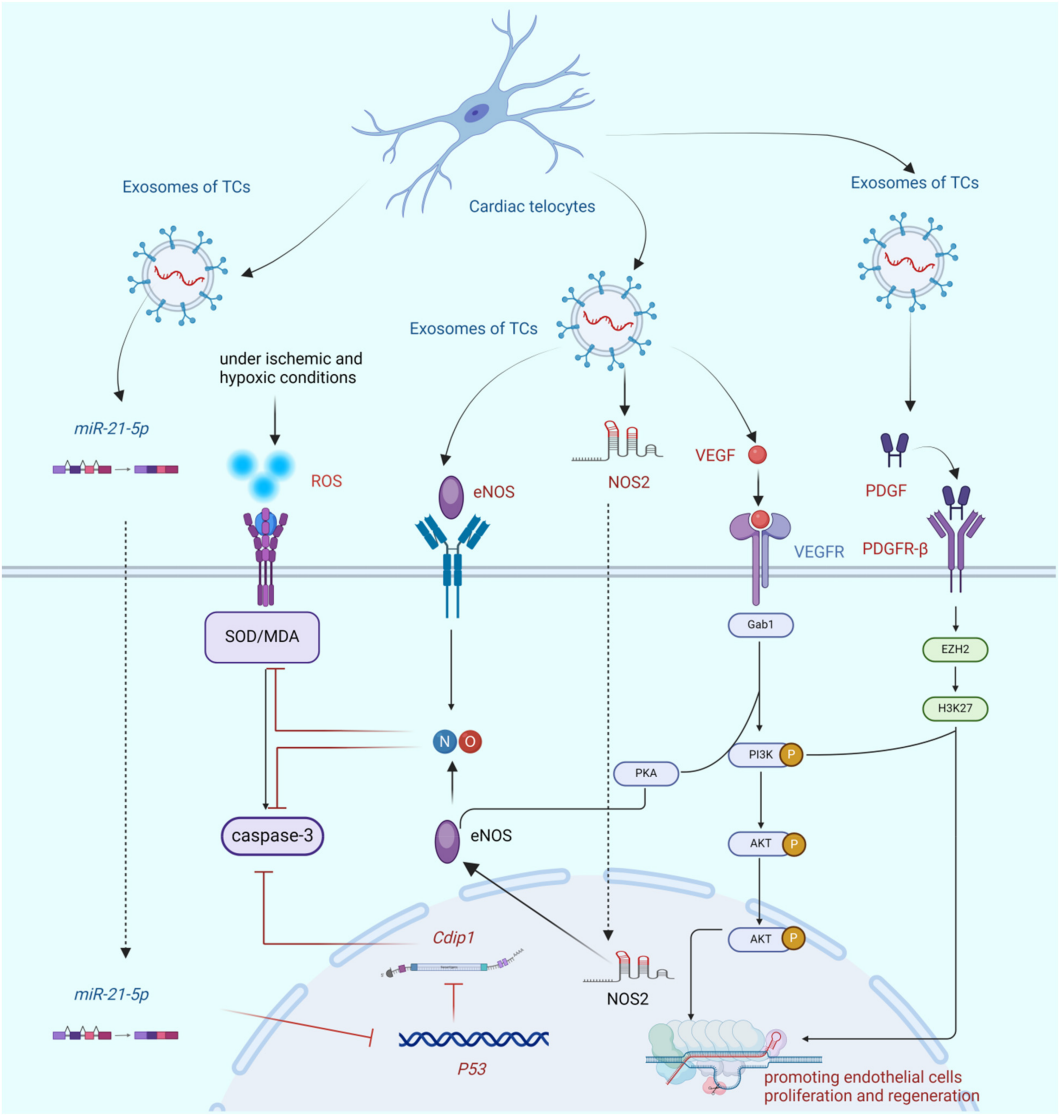
The bio-mechanism of cTCs promoting angiogenesis. This figure demonstrates that cTCs inhibit endothelial cell apoptosis via paracrine secretion of miR-21-5p and subsequent regulation of the P53/Cdip1 pathway. Additionally, cTCs secrete paracrine factors eNOS and NOS2, which mitigate endothelial cell apoptosis and ameliorate oxidative stress by augmenting NO synthesis. Furthermore, cTCs-derived paracrine VEGF not only stimulates endothelial cell proliferation but also enhances NO production. Finally, paracrine PDGF released by cTCs promotes endothelial cell proliferation through activation of the PI3K/AKT signaling cascade. (Cdip1, Cdip1 gene; SOD, superoxide dismutase; ROS, reactive oxygen species; eNOS, endothelial nitric oxide synthase; NO, nitric oxide; NOS2, nitric oxide synthase 2; VEGF, vascular endothelial growth factor; Gab1,Grb2 associated binding protein 1; PI3K/AKT, phosphatidylinositol 3 kinase protein kinase B; PKA, protein kinase A; PDGF, platelet-derived growth facter; PDGFR-β, platelet-derived growth factor receptor; EZH2, histone-lysine N-methyltransferase; H3K27, histone H3 trimethylated lysine 27).

Studies have demonstrated that the number of cTCs in the region affected by acute myocardial infarction is significantly reduced. Studies have substantiated that, subsequent to the induction of myocardial infarction (MI) via ligation of the left anterior descending coronary artery (LAD), the synchronous intramyocardial injection of 10⁶ cTCs into both the infarcted myocardial region and the peri-infarct border zone can attenuate the infarct size, foster neovascularization, mitigate myocardial fibrotic remodeling, and ameliorate cardiac function 14 weeks after the intervention. This treatment may also improve cardiac function and reduce myocardial fibrosis (20, 61). The study demonstrates that cTCs can generate several microRNAs, which are well-known to play crucial regulatory roles in promoting the growth, migration, and angiogenic activity of endothelial cells. These microRNAs include miR-let-7e, miR-10a, miR-27b, miR-100, miR-126-3p, miR-130a, miR-143, miR-155, and miR-503 (22). Studies have verified that miR-25-5p, secreted by cTCs, can impede endothelial cell apoptosis. It achieves this by precisely targeting and silencing the cell death-inducing P53 target 1 (Cdip1) in coronary microcirculation endothelial cells. This mechanism is likely to be pivotal in the promotion of angiogenesis (23). During the neovascularization phase of acute myocardial infarction in rats, the number of cTCs in the border area of myocardial infarction increases significantly. Moreover, a large number of vesicles secreted by cTCs can be observed in the matrix between cTCs and endothelial cells. This study has identified significant changes in the number of cTCs after myocardial infarction. However, the reasons for the increase in the number of cTCs are still unclear. It remains unknown whether cTCs are migrating from the blood similar to inflammatory cells or if the signal transduction associated with myocardial cell infarction is stimulating the proliferation of cTCs. These cTCs and vesicles exhibit a high expression of VEGF and NOS2, indicating that cTCs may promote the formation of blood vessels in the border area of myocardial infarction by secreting vesicles (22). Cluster of differentiation 31(CD31), VEGF, PDGFR-β, and kruppel-like factor 4(KLF-4) were highly expressed (27, 86, 91). VEGF is widely acknowledged as the paramount factor in promoting angiogenesis, whereas PDGFR-β plays a crucial role in maintaining the stability of cardiac angiogenesis. Additionally, KLF-4 is a crucial regulatory factor that promotes endothelial cell proliferation and differentiation. Based on these factors, it can be inferred that cTCs, which produce these factors, play a crucial role in sustaining the growth of blood vessels in the heart, as shown in Figure 3.

### 5.2 The mechanism of anti-thrombotic effect of cTCs

Thrombosis is a significant mechanism that leads to IHD. The exocrine secretion of cTCs contains various miRNAs that regulate the thrombosis process at different targets. It is a glycoprotein on the surface of the endothelial cell membrane. Studies have demonstrated that miR-92a has the ability to enhance the expression of Kruppel-like factor 2 (KLF2) and TM in human uveal epithelial cells through its binding to KLF2. Therefore, the down-regulation of miR-92a may be connected to thrombosis (92, 93). The levels of miR-21, miR-409-3p, miR-432, and miR-150 were significantly lower in patients with atrial fibrillation compared to the control group. Nonetheless, subsequent to radiofrequency ablation, the levels of these microRNAs significantly increased, indicating that they may possess a pivotal role in the process of thrombosis (94–96). The expression of miR-126 was significantly decreased in acute myocardial infarction, which is one of the markers of endothelial cell injury. This suggests that the reduced expression of miR-126 may increase the risk of thrombosis (97, 98). Tissue factor (TF) is a promoter of the exogenous coagulation pathway. However, miR-451a inhibits the expression of coagulation factors such as TF, factor X coagulant (FXa), and von willebrand factor (vWF) by targeting the IL6R/STAT/TF pathway. Simultaneously, miR-451a significantly up-regulates the expression of anticoagulant factors such as antithrombin III (ATIII), tissue factor pathway inhibitor (TFPI), and tissue plasminogen activator (tPA). This helps to suppress the formation of intravascular thrombosis. Therefore, miR-451a is one of the important miRNAs that inhibit thrombosis (99). Thromboxane A2 (TXA2) promotes platelet aggregation while prostaglandin-I-2 (PGI2) inhibits it. The balance between TXA2 and PGI2 is crucial in preventing both thrombosis and bleeding. Research has indicated that the expression of miR-26a-5p is significantly decreased in patients with coronary heart disease. Upon treatment with a miR-26a-5p inhibitor, the serum levels of TXA2, endothelin-1 (ET-1), and angiotensin II (Ang II) were upregulated, whereas the expression of PGI2 and eNOS were downregulated. Consequently, this led to reduced endothelial cell activity and increased apoptosis. The mechanism behind this is related to the inhibition of the PI3K/AKT signal. Therefore, miR-26a-5p expression is a crucial factor in thrombosis (100). However, this study was unable to identify drugs or key regulatory factors that upregulate the expression of miR-26a-5p in cTCs, thereby lacking more convincing evidence for the inhibitory effect of cTCs on thrombosis. According to the study, during transcription, the expression of miR-301a/miR-454 is downregulated by the co-regulation of hypoxia-inducible factor-1α (HIF-1α) and peroxisome proliferator-activated receptor-α (PPAR-α). The low expression levels of miR-301a/miR-454 can lead to an increase in endothelin-1 (ET-1) and plasminogen activator inhibitor-1 (PAI-1), suggesting that miR-301a/miR-454 may play a regulatory role in thrombosis (101).

### 5.3 The inhibition mechanism of cTCs on inflammation

**Figure 4:**
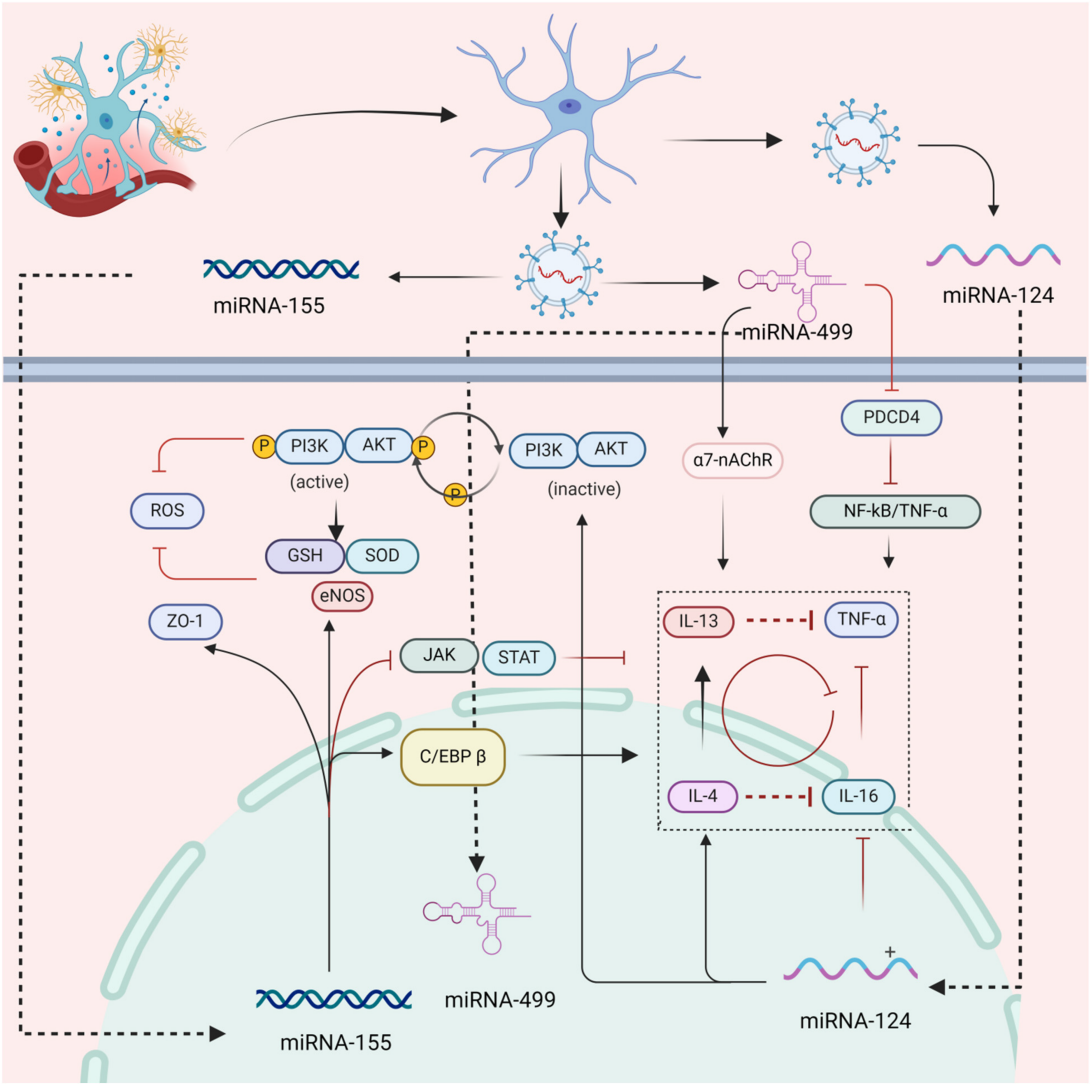
The mechanism of inhibition of inflammatory of cTCs. This figure demonstrates that cTCs upregulate the expression of ZO - 1 via paracrine secretion of miR - 155, which in turn elevates vascular permeability. Simultaneously, cTCs upregulate the expression of eNOS to suppress oxidative stress and inhibit the JAK/STAT pathway, thereby alleviating the inflammatory response. Additionally, cTCs secrete miR - 499 paracrinely to upregulate α7-nAChR and downregulate the expression of PDCD4, significantly suppressing the inflammatory response. Furthermore, paracrine miR - 124 released by cTCs can directly inhibit the expression of inflammatory factors such as IL - 4 and IL - 16. (ZO-1, zonula occludens protein 1; eNOS, endothelial nitric oxide synthase; C/EBP β, enhancer-binding proteins β; ROS, reactive oxygen species; GSH, glutathione; SOD, superoxide dismutase; PI3K/AKT, phosphatidylinositol 3 kinase protein kinase B; 7-nAchR, α7 nicotinic acetylcholine receptor; PDCD4, programmed cell death protein 4; TNF-α, tumor necrosis factor-α; NF-ΚB, nuclear factor-κB; JAK/STAT, Janus kinase/signal transducers and activators of transcription;IL-4, interleukin-4; IL-16, interleukin-16; IL-13, interleukin-13).

MiR-130a, miR-124, miR-155 and miR-499 are thought to play crucial roles in regulating the permeability of microcirculatory vessels, the formation of edema, and the expulsion of inflammatory factors. Exosomes secreted by cTCs are rich in this miRNA, which allows them to regulate the barrier function of microvasculature by transferring these miRNAs to endothelial cells. Studies have shown that miR-130a, produced by endothelial cells exposed to ischemia, disrupts the integrity of the blood-brain barrier. This occurs through the regulation of the transcription factor homeobox A5 (HOXA5), which is related to the expression of the tight junction protein occludin. However, the administration of a miR-130a antagonist can improve the permeability of the blood-brain barrier and lead to edema in brain tissue. Consequently, miR-130a is a factor that can cause enhanced microvascular permeability (102, 103). miR-124 has a specific effect on inhibiting exudation and inflammatory reactions. It functions as an anti-inflammatory agent by regulating the IL-6/TNF-α signal transduction process. Additionally, its expression is up-regulated by the anti-inflammatory factors IL-4 and IL-13. However, miR-124 can also increase the production of reactive oxygen radicals by inhibiting PI3K/AKT signal transduction, which can lead to the apoptosis of endothelial cells (104). Therefore, miR-124 may have a dual impact on IHD. On one hand, it can reduce inflammatory exudation and suppress the inflammatory response, on the other hand, it may accelerate the damage to endothelial cells (105). Research has shown that miR-155 plays a crucial role in regulating microvascular barrier function and inflammatory response. By regulating the expression of tight junction protein zonula occludens-1 (ZO-1) and eNOS in endothelial cells, specific inhibitors of miR-155 can help to maintain microvascular barrier function and regulate vascular diameter (106, 107). The study revealed that the inhibition of miR-155 expression has varying impacts on the regulatory function of the inflammatory response over time. In the early stage (7 days), inhibiting JAK/STAT signal transduction can suppress the inflammatory response. In the late stage, miR-155's key target, CCAAT/enhancer binding protein β (C/EBP β), is up-regulated to enhance the inflammatory response, which facilitates the removal of necrotic tissue and promotes tissue repair (108, 109). miR-499 is an important marker of myocardial injury, and its expression is closely related to the prognosis of cardiovascular disease. Studies have found that the programmed cell death 4(PDCD4) protein can inhibit the NF-kB/TNF-α signal, reducing the inflammatory response. However, miR-499 counters this effect by targeting PDCD4, inducing inflammatory damage to endothelial cells (110). Studies indicate that the α7 nicotinic acetylcholine receptor (α7-nAchR) is expressed by endothelial cells and functions as a cholinergic anti-inflammatory pathway regulator, inhibiting the release of inflammatory factors (111–113). In acute myocardial infarction, endothelial cells internalize miR-499 and target α7-nAChR, significantly enhancing the inflammatory response of endothelial cells (114). The following miRNAs play a regulatory role in vascular inflammation: miR-146a, miR-217, miR-34a, miR-126, miR-21, miR210, and miR-181b (115, 116). It should also be noted that in addition to miRNA these mtDNA in cTCs were often released into the microenvironment and played an important role in the development of different types of inflammatory diseases, including acute and chronic ischemic diseases, traumatic brain injury, and infectious diseases (117). cTCs exocrine secretion contains multiple miRNAs that can regulate microvascular inflammatory exudation, playing an intervention role in IHD, as shown in Figure 4.

### 5.4 The mechanism of cTCs regulating microvascular diastolic and systolic function

Vascular diastolic and systolic function disorders are major contributors to IHD. ET-1, Angiotensin II (Ang II), vasopressin, and epinephrine are vasoconstrictive substances, while NO, prostacyclin (PGI2), endothelial hyperpolarizing factor (EDHF), and bradykinin have vasodilatory effects. Currently, ET-1, NO, PGI2, and EDHF are considered crucial regulators of endothelium-dependent vasodilation or vasoconstriction dysfunction, and their secretion and formation are closely linked to endothelial cell function. Research indicates that the upregulation of miR-21a-3p expression plays a significant role in vasodilation, and that silencing miR-21a-3p expression can markedly reduce the expression of eNOS, a crucial factor in affecting NO synthesis in endothelial cells (118). The study discovered that miR-21 can impact the expression of both ET-1 and eNOS by regulating the MAPK/ET-1 and PTEN/AKT/eNOS signal pathways. This suggests that miR-21 plays a role in controlling vasodilation and contraction functions by affecting the downstream targets of these pathways (119, 120). The expression of miR-92a is positively correlated with the level of ET-1, while the expression of miRNA-1 is negatively correlated with ET-1 (121). This indicates that maintaining a balance between miR-92a and miRNA-1 expression is vital for vasoconstriction. Although this study implies that maintaining the balance of miR-92a/miRNA-1 is crucial for regulating the expression of ET-1, a key regulator of vasodilation, the effectiveness of this balance could not be assessed *in vivo* using more objective methods such as magnetic resonance imaging techniques. Moreover, the study failed to propose key factors that regulate the balance of miR-92a/miRNA-1. Research has shown that overexpression of miR-124 and miR-34c can increase PGI2 expression, resulting in vasodilation. This mechanism is related to the regulation of the JAK2/STAT3 signal pathway (122).

### 5.5 The mechanism of cTCs inhibiting of vascular remodeling

The main pathological changes that occur during vascular remodeling are caused by the deposition of collagen fibers and the reduction of vascular smooth muscle cells. In this process, cTCs express type IV collagen, matrix metalloprotein-3, -9, -10 (MMP-3, -9, -10), which play a crucial role in the formation and degradation of the vascular basement membrane. Type IV collagen is the most important component of the vascular basement membrane (59, 123). According to the study, the skin tissue of psoriasis patients showed significant damage to their microvascular structure, and there was a considerable reduction in the number of TCs. However, after effective treatment, the vascular remodeling significantly improved, and the number of TCs increased, indicating that TCs play a vital role in the regulation of vascular remodeling (124). The study found that miR-197-3p can inhibit extracellular matrix degradation and increase vWF expression by targeting tissue inhibitor of metallo proteinase 3 (TIMP3), thus promoting vascular remodeling and thrombosis (125, 126). Studies have indicated that miR-21a-3p in cTCs increases expression of matrix metalloproteinase-2 (MMP-2) by activating the PI3K/AKT/mTOR pathway. MMP-2 is a vital factor in the degradation of the vascular basement membrane (118). The study revealed that miR-92a downregulated alpha-smooth muscle actin (α-SMA), smoothelin (SMTN), and calponin (CALP), while upregulating fibronectin (FN), osteopontin (OPN), and thrombospondin (TSP). These factors play a crucial role in the phenotypic transformation of vascular smooth muscle cells and are important contributors to the acceleration of arteriosclerosis (127). Signal transduction inhibitor 1(SOCS1) functions as a cytokine signaling inhibitor capable of negatively modulating the JAK/STAT signaling pathway. MiR-155-5p activates the JAK2/STAT3 signaling pathway through the inhibition of SOCS1 expression, subsequently promoting the expression of vascular endothelial growth factor A (VEGFa), matrix metalloproteinase-9 (MMP9) and fibroblast growth factor 2 (FGF2). This regulatory effect plays a crucial role in processes such as inflammatory responses, immune responses, and vascular remodeling (128).

## 6 The protective effect of cTCs on cardiovascular diseases

The study discovered that patients with heart failure, myocardial infarction, cardiac systemic sclerosis, and myocardial fibrosis had significantly reduced numbers of cTCs and their Tps. It was also observed that the number of cTCs gradually decreased with age, and an imbalance between the death and proliferation of cTCs could lead to severe myocardial fibrosis (16, 18, 52, 129, 130). Although the reasons behind the decrease in the number of cTCs are not fully understood, it is believed that ischemia, oxidative stress, fibrosis, and amyloid changes are closely related to this phenomenon. The number of cTCs in the early infarction area and its surrounding region significantly decreased, while the number of cTCs in the surrounding area of MI increased significantly during the neo-vascular phase after myocardial infarction (61). A study focusing on that issue involved transplanting cTCs into the myocardium within 30 min after induced MI. The transplantation was carried out by administering three injections at the MI boundary area and two injections at the center of the ischemic zone. It is imperative to evaluate the outcomes 14 weeks after the MI (20). Surprisingly, transplanting cTCs into the infarcted myocardium resulted in a reduction in infarct size, enhanced myocardial function, and ultimately reduced diastolic and systolic pressure. Furthermore, increased angiogenesis stimulated normal reconstruction of the left ventricle without excessive fibrosis. Compared to the control group, higher left ventricular thickness was observed in the ischemic and border areas, and lower collagen area in the infarcted area. Hence, it is feasible to partially reverse the consequences of MI. It is currently believed that cTCs can regulate the function of endothelial cells and angiogenesis by secreting various angiogenic factors, such as VEGF and different miRNAs. The occurrence of MI can result in a significant reduction in the cardiac microvascular network, which is one of a significant characteristic of IHD. cTCs can promote the formation of microvasculature and improve blood perfusion after infarcted myocardium, proving that they can be used for the prevention and treatment of IHD.

Studies have shown that the accumulation of oxygen free radicals induced by oxidative stress can alter the expression of many genes in cTCs and cause cell death, which can accelerate the onset of myocardial remodeling and myocardial fibrosis (131, 132). An increase of fibrillary collagens due to replacement fibrosis is connected to a reduction in cTCs and Tps quantity or even their absence (133). Similarly, that myocardial fibrosis can have a reverse effect on the proliferation of cTCs and can restrict the extension of Tps (19, 129, 134). The impact of various extracellular matrix proteins on cTCs varied significantly. Fibronectin exhibited the strongest inhibitory effect on cTCs proliferation and Tps extension, while laminin had the weakest effect (135). During heart failures, the cytoplasm of cTCs undergoes obvious ultrastructural changes, which include vacuolation, loss of the reticular fibrous skeleton, and shortening or disappearance of Tps. These changes are caused by increased levels of inducible nitric oxide synthase (iNOS), cyclooxygenase-2 lipid peroxide, andestradiol, leading to local inflammation or ischemic microenvironment (136). cTCs are linked to myocardial amyloidosis and can limit its spread by surrounding and restricting amyloid fibers, which can aid in treating atrial fibrillation (137, 138). It is believed that cTCs can assist in alleviating fibrosis as they communicate with fibroblasts and myofibroblasts. However, the exact mechanism of this interaction remains unclear (14). There is a theory suggesting that the loss of cTCs and Tps leads to an enhanced conversion of fibroblasts to myofibroblasts and the further spread of elastin and collagen fibers within systemic sclerosis tissue (51). The hypothesis that cTCs promote the conversion of fibroblasts to myofibroblasts may also be related to the fact that cTCs enhance the transformation of vascular smooth muscle cells (VSMCs) into myofibroblasts, thus accelerating vascular remodeling. Vascular remodeling is one of the other pathological changes observed in IHD.

The decrease in the number of cTCs is closely related to the occurrence of heart failure. Moreover, cTCs display cytoplasmic vacuolisation, shrinkage of Tps, and a loss of characteristic labyrinthine components. These changes occur in conjunction with an inflammatory or ischemic micro-environment in the myocardial tissue following heart failure, which is rich in iNOS, cyclooxygenase-2, and lipid peroxide (130). Additionally, TUNEL labeling demonstrated an imbalance between the proliferation and apoptosis of cTCs, with apoptosis being predominant (133). Moreover, there is a positive correlation between the number of cTCs in heart tissue and the number of denatured collagens or non-fibrillar collagens, as well as with advancement of acute or chronic myocardial inflammation. As cTCs are vital to intercellular signalling in human heart, decrease in their number may lead to important disturbances in bioelectrical communication and contribute in arrhythmogenesis (51). There exist multiple mechanisms which cTCs are implicated in the occurrence of arrhythmia. Firstly, the distribution of cTCs in the sleeves of pulmonary veins has an impact on the electrical activity rhythm of myocardial cells (139). Secondly, cTCs have an impact on the accumulation of amyloid-like substances in myocardial cells. Thirdly, cTCs have an impact on the acute and chronic inflammatory responses of myocardial tissue (140). If these mechanisms can also be confirmed between cTCs and endothelial cells and VSMCs, it will show that cTCs can treat IHD by inhibiting the inflammatory response, improving vascular remodeling, and controlling vasodilation and contraction.

## 7 The limitations of cTCs research

Currently, the understanding of the morphological characteristics and surface markers of cTCs remains somewhat unclear. Different research teams may employ diverse criteria for defining and identifying cTCs, thereby affecting the comparability of research findings. cTCs play multiple roles in both physiological and pathological processes, including intercellular communication, tissue repair, and regeneration. However, their specific functional mechanisms have not yet been fully elucidated. cTCs are relatively rare in tissues and are intermingled with other cell types, which complicates their isolation and purification processes. Current isolation methods often demand a substantial investment of time and effort, and it is challenging to obtain highly purified TCs reliably. Additionally, the *in vitro* culture conditions for TCs have not been fully optimized. During the culture process, issues such as reduced cell viability and altered cellular functions may arise. Although cTCs have been implicated in the occurrence and development of various diseases, further in-depth investigations are necessary to fully understand their specific roles and mechanisms in different disease contexts. At present, most studies on cTCs therapy are still in the animal experimentation or early clinical trial phases. There is a lack of comprehensive evaluation regarding their long-term efficacy and safety. Moreover, the treatment regimens for cTCs therapy, including aspects such as the routes of cell administration, dosage, and timing, remain uncertain and require further refinement. Furthermore, the research and application of cTCs raise several ethical considerations, such as the source of cells, obtaining informed consent from patients, and ensuring patient privacy protection.

## 8 Conclusions

Over the years, there has been significant progress in treating cardiac and large vessel occlusive diseases due to the extensive development of cardiac interventional therapy. Nevertheless, the neglect of myocardial hypoperfusion resulting from coronary microvessel injury has a significant impact on the clinical management of IHD. While a lot of studies have been carried out on the positive effects of cTCs on various cardiovascular diseases like MI, heart failure and atrial fibrillation. The therapeutic effect of cTCs on IHD is still relatively unknown. Although some miRNAs mentioned in the article are only referred to in the exocrine of cTCs, various clues suggest that they may have a positive effect on IHD. Most of the current research is focused on studying the paracrine mechanism of active substances released from cTCs, while there are few studies on the importance of the direct contact mode between cTCs and endothelial cells. The direct contact mode between cTCs and endothelial cells may also produce unexpected positive effects. Therefore, the direct contact mode between cTCs and endothelial cells should also be the focus of future studies.

In summary, we believe that the paracrine effect of cTCs can improve the function of vasculature by promoting angiogenesis, reducing thrombosis, regulating vasodilation and contraction, inhibiting inflammatory exudation, and vascular remodeling. cTCs play an irreplaceable role in the treatment of IHD, so it is necessary to study the systematic mechanism of cTCs. In addition, our upcoming research will concentrate on the exploration of other non-coding RNAs and additional bioactive proteins within cTCs or their exosomes. This exploration will be conducted employing advanced genomic technologies, high-throughput microarray platforms, and comprehensive proteomic approaches. Subsequently, the identification and confirmation of the key bioactive substances will be achieved through precise *in situ* hybridization staining techniques, both in cellular contexts and in rat heart tissue samples.

## Data Availability

The original contributions presented in the study are included in the article/Supplementary Material, further inquiries can be directed to the corresponding author.
